# Outcomes of a 5-week individualised MDT outpatient (day-patient) treatment programme for functional neurological symptom disorder (FNSD)

**DOI:** 10.1007/s00415-020-09874-5

**Published:** 2020-05-14

**Authors:** Panayiota Petrochilos, M. S. Elmalem, D. Patel, H. Louissaint, K. Hayward, J. Ranu, C. Selai

**Affiliations:** 1 Department of Neuropsychiatry, National Hospital for Neurology and Neurosurgery, Queen Square, Box 19, London, WC1N 3BG UK; 2 Department of Clinical and Movement Neurosciences, National Hospital for Neurology and Neurosurgery, UCL Queen Square Institute of Neurology, Queen Square, Box 95, London, WC1N 3BG UK; 3 Therapies Services Department, National Hospital for Neurology and Neurosurgery, Queen Square, London, WC1N 3BG UK

**Keywords:** Functional neurological symptom disorder, Functional neurological disorders, Conversion disorder, Psychogenic, Dissociative disorders, Multidisciplinary team, Therapy, Outpatient

## Abstract

**Aim:**

We report results from a 5-week MDT treatment programme, with individualised sessions, for a selected group of patients with FNSD, delivered in a neuropsychiatric outpatient setting. Primary aims were to (1) reduce symptoms, (2) improve functional performance and (3) improve health status.

**Methods:**

Treatment involved individual sessions of neuropsychiatry, cognitive behavioural therapy, physiotherapy, occupational-therapy, education and family meetings. Outcome measures collected at the beginning and end of treatment and at 6 months, were patient and clinician reported. Aims were assessed by the following: symptom reduction (PHQ15, PHQ9, GAD7, SPIN, Rosenberg); health and social functioning (HONOS, WSAS); functional performance (COPM); health status (EQ-5D-5L) and patient-rated perception of improvement (CGI).

**Results:**

Analyses of 78 patients completing the programme and attending a 6-month review revealed high-baseline levels of disability compared to EQ-5DL population norms and high rates of disability and psychopathology as indicated by the WSAS and mental health indices (PHQ9, GAD7, SPIN, Rosenberg’s self-esteem). At baseline, 92.3% met the IAPT caseness threshold for depression and 71% met the IAPT caseness threshold for anxiety. A Friedman ANOVA over the three time points and Dunn-Bonferroni post hoc tests indicated statistically significant improvements from admission to discharge and admission to 6-month follow-up. Sustained improvements were seen in somatic symptoms (PHQ15), depression (PHQ9), anxiety (GAD7), health and social functioning (HONOS), functionality (COPM), health status (EQ-5D-5L) and patient-rated clinical global improvement (CGI).

**Conclusion:**

An MDT can effectively deliver an outpatient programme for FNSD which can serve as an alternative to costlier inpatient programmes. Early identification and treatment of co-morbidities is advised.

**Electronic supplementary material:**

The online version of this article (10.1007/s00415-020-09874-5) contains supplementary material, which is available to authorized users.

## Introduction

Functional neurological symptom disorders (FNSD) [1] encompass symptoms seemingly manifested through the nervous system, but which are not caused by a physical neurological disease. Other names include psychogenic, psychosomatic, somatization, medically unexplained symptoms and conversion disorder. The current preference, following the DSM-5 adoption of the term functional is intended to be causally neutral [2]. Although the requirement to identify an associated psychological factor was removed from the criteria in DSM-5, the importance of exploring psychological stressors continued to be emphasised in the accompanying text [3]. In the ICD-11, it is referred to as dissociative neurological symptom disorder [4].

FNSD accounts for approximately 6% of neurology outpatient contacts and community incidence rates of 4–12 per 100,000 per annum [5]. The diagnosis is considered reliable, with revision rates less than 5% [5].

There are many different symptom types ranging from those impairing movement (e.g., weakness, dystonia, jerks), sensation (e.g., tingling, pins and needles), dissociative episodes and those impairing bladder, bowel, vision, swallowing, speech and cognitive functioning [5, 6]. Symptoms can fluctuate in duration from brief and episodic to more prolonged and persistent.

Comorbid neurologic disease occurs in around 10% of cases [5]. Psychological comorbidity rates are consistently higher than comparable neurologic disorders, with rates of depression between 20 and 40% [7, 8, 9]. High rates of anxiety (e.g., 38%, [10]) and high rates of panic symptoms have been reported in patients with dissociative seizures [11, 12, 13]_._ Personality disorders have been reported with rates of 45% in functional movement disorders and similar rates in dissociative seizures [14].

Levels of disability can vary, be complicated by pain and fatigue and be accompanied by high rates of unemployment [5, 13].

Treatments can include early intervention with neurology and psychiatry working together [15], focusing on specific symptoms such as cognitive behavioural therapy (CBT) for dissociative episodes [14, 16], physiotherapy for functional movement disorders [17, 18] or 1-week multidisciplinary (MDT) programmes for functional movement disorders (physical, occupational, psychotherapy, SALT) [19, 20]. More complex and heterogenous symptom presentations often with co-morbidities and high levels of disability, have been referred to MDT (multidisciplinary team)-based programmes which have been delivered in inpatient neuropsychiatric settings [21, 22].

## Aim

We aimed to assess whether a 5-week outpatient-based MDT treatment programme for FNSD (including neuropsychiatry, cognitive behavioural therapy (CBT), physiotherapy, occupational therapy (OT), previously shown to have sustained long-term benefit when delivered as an inpatient programme [21], could be delivered effectively in an outpatient setting and demonstrate sustained improvements. The primary aims of the programme were to (1) reduce symptoms, (2) improve functional performance and (3) improve health status.

## Methods

### Referrals

Referrals were accepted from consultant neurologists and GPs on advice of neurologists following a prior diagnosis of FNSD. Presentations included functional movement symptoms, functional sensory symptoms, non-epileptic/dissociative symptoms and combinations of these.

### MDT assessment clinics triage

277 patients were seen in multidisciplinary assessment clinics running over a 15-month period, to assess suitability for participation in any treatment at NHNN (see Supplementary material Table 1). Inclusion criteria were (1) patient-identified need(s) for treatment, (2) agreement with diagnosis, (3) understanding of diagnosis translatable into functional goals, (4) readiness to engage with treatments provided including within a neuropsychiatric service and use of a CBT-based model and (5) predominant need not better met by an alternative service. Outcomes of this clinic were based on clinical decision, and agreed collaboratively with the patient following discussion and explanation. These included either: (1) participation in the new 5-week outpatient programme (39%) [advised if physiotherapy, OT and CBT needs and able to tolerate the commute or stay with a carer in a hotel, or unable to tolerate the inpatient environment], (2) participation in an established 4-week inpatient programme (22%) [advised if physiotherapy, OT, CBT and nursing needs including need for medication administration, comorbidities, hoist transfer, unable to tolerate the outpatient commute or additional supportive function required], (3) Outpatient CBT (6%) [advised if able to work with the CBT model, predominate NES, or few OT/physiotherapy needs] or (4) another outcome (16%). 17% were discharged as they did not attend the initial assessment (see Supplementary material Table 1). Exclusion criteria were (1) acute mental health crisis, (2) pain or fatigue of a degree impairing participation in programme at current point.

### Intervention

The outpatient programme ran over 2 days a week for 5 weeks. This was led by the neuropsychiatry service at the National Hospital for Neurology and Neurosurgery. The setting was in the general outpatient clinic area with access to rehabilitation gyms and therapy kitchen facilities. Patients living outside of London were accommodated in a nearby hotel close to the hospital with the option of a relative/carer staying with them. There were four patients in each cohort running over 2 days.

Overall, the programme included a group education session to build up an understanding of the diagnosis, a goal-setting session followed by individual treatment sessions of CBT (× 9), physiotherapy (× 9), OT (× 9), consultant neuropsychiatry sessions (× 3) and a family session.

### MDT Model for functional neurological symptoms

The multidisciplinary team (MDT) model used, was an integrated approach focused on individualising care for a complex condition.

Common to all treatment modalities was a collaborative approach. The patient was actively engaged in diagnostic explanation and education to gain confidence in their diagnosis, thereby facilitating formulation, goal setting and identification of triggers and perpetuating factors. Patients were encouraged to use a therapy workbook and were supported to develop a relapse prevention plan.

The first day of initial assessments focused on formulation of difficulties primarily within a CBT framework focused on predisposing, precipitating and perpetuating factors. Perpetuating factors could then be addressed at a cognitive, behavioural and systemic level across treatment modalities. The second day involved a group education session to which family members were also invited. This covered pathophysiological explanations relevant to FNSD, symptom formulation including triggering factors, the disruptive potential of self-focused attention, anticipation, the stress-response cycle, maintaining factors such as safety behaviours and unhelpful reinforcement of symptomatic movement patterns. These were mapped on to a CBT-based model which would form the foundation of treatment.

Where appropriate, therapists gave joint sessions combining disciplines to augment effect and facilitate transfer of concepts and skills across different domains of functioning.

### Neuropsychiatry

There were three sessions with a neuropsychiatrist in the form of assessment and two progress meetings. The role of the neuropsychiatrist is crucial for reviewing the diagnosis, considering co-morbidities, supporting education and explanation, initiating pharmacological treatment where appropriate, reducing unnecessary medications, exploring barriers to progress and assisting with appropriate onward referrals.

### Cognitive behavioural therapy (CBT)

The 9 (out of 12) sessions with the CBT therapists included a personalised explanation of the CBT model through formulation based on identification of the patient’s own predisposing, precipitating and perpetuating factors. The aim was to build insight and awareness into emotions and triggers and make links with behaviours perpetuating maladaptive symptoms or responses to certain situations/states. Behavioural interventions were used between sessions to challenge avoidance and safety behaviours to develop alternative ways of responding and reacting to triggers.

Emergent themes specific to individuals such as assertiveness, perfectionism and heightened sense of responsibility were explored. Other tasks involved emotional processing of unprocessed issues, acceptance of diagnosis and working on thoughts/cognitions, shifting perspective and identifying when individuals fell into unhelpful thinking patterns. Techniques included positive data logging, journaling and problem solving. Work was reinforced by documentation of progress in a therapy workbook.

### Occupational therapy (OT)

OT for FNSD on the programme aimed to assist patients to engage in daily activities that they had been unable to do or had found difficult since the onset of symptoms. The aim was to normalise participation and thus reduce reliance on the use of equipment and input from others. Self-management principles and the use of graded goal setting were central. Sessions were focused on identifying barriers to participation and integrating education and symptom management techniques into function with daily activities. Interventions included: assistance to manage fatigue, pain and anxiety, improving structure and routine, grading and practising daily activities (e.g., cooking), exploring how cognitive challenges could be reduced, improving confidence and independence with accessing the community and exploring return to vocational roles (work, education, childcare, volunteering and leisure).

### Physiotherapy

Physiotherapy for movement disorders was focused on movement re-training aiming to restore normal movement during problematic activities [23, 24]. Goals were set and positive signs demonstrating the potential for normal movement were elicited. Once simple movements were achieved, complexity was increased. Movement retraining was accompanied by distraction of self-focus with attention focused alternatively on cognitive activities or task-based activities and using symptom-specific strategies. A CBT model was used to challenge beliefs about the assumed consequences of movement and use of compensatory strategies to modify the efficiency of movement [24]. Where applicable, there was a focus on improved understanding of pain, movement and exercise and initiating a graded exercise approach to extend physical capacity.

### Goal setting for the next 6 months

In the last week, all disciplines discussed relapse prevention plans and collaboratively set goals for patients to work towards over the coming months and to be reviewed at the 6-month face-to-face follow-up. The therapy workbook was reviewed, summarising the patient’s understanding of the problem, triggers, warning signs (e.g., withdrawal/avoidance), techniques they found most useful and their plan to maintain progress alongside a relapse prevention plan.

### Family meeting

A family meeting at the end of the programme, was a space for patients to reflect with family/carers on their progress. This included reviewing changes in their symptoms, mood, day to day function, goals set at the beginning of treatment and the goals they wanted to work on over the next 6 months. It highlighted things the family could continue to work on to support the patient and to address any maintaining factors such as overprotective behaviours. It facilitated a degree of emotional processing, reflection of issues and considering how roles in the family may have changed.

### 6-month review

A 6-month face-to-face follow-up with the patient and MDT team facilitated review of progress, 6-month goals and measurement of outcomes.

### Outcome measures

Outcome measures (described in Supplementary material Table 2) were collected at the start and end of the programme and at the 6-month review. These included: clinician-rated outcome measures: Health of the Nation Outcome Scales (HONOS) [25] and patient rated: somatic symptoms (PHQ15) [26], Patient Health Questionnaire (PHQ9) [27], generalised anxiety (GAD7) [28], Rosenberg self-esteem [29], social phobia inventory SPIN [30], EQ-5D-5L [31], Canadian Occupational Performance Measure (COPM) [32], Work and Social Adjustment Scale (WSAS) [33] and the Clinical Global Impression (CGI) [34] and a benefit of programme visual analogue scale. The question asked ‘please place on the horizontal line where you feel best represents how much you benefitted from this programme’. This line was 10 cm long and labelled from ‘very little’ to ‘a great deal’ where 1 cm is 10%.

### Analysis

Statistical analysis of outcome measures was performed with SPSS version 22. As data were not normally distributed a Friedman ANOVA was conducted on median scores as summarised in Table 2. Post hoc comparisons were evaluated with a Dunn Bonferroni test and effect sizes were analysed with a Kendall’s W. The study was approved as a service evaluation by the departmental audit lead and registered with the quality and safety forum of University College Hospital NHS Foundation trust. As such, it did not require ethics committee approval.

## Results

Data were collected between March 2017 and August 2018. During this period, 106 consecutive patients with FNSD were invited to attend the programme. 3 failed to attend on the first day and of these, two were uncontactable and one cited childcare difficulties. 3 dropped out after having started: one left 2 days after assessment having already almost recovered requesting further psychotherapy; one left after a week and was uncontactable; and one left after 3 weeks, citing no benefit and wanting to pursue musculoskeletal physiotherapy and hydrotherapy.

100 patients started and completed the 5-week programme. 3 were excluded from the analysis as they were not able to complete their outcome measures at the end of the 5-week programme. 19 were excluded from the analysis as they did not attend their 6-month review and outcome measures were not available. This group had a larger proportion of men and overall marginally less severe baseline scores on PHQ15, SPIN, self-esteem and WSAS (see Supplementary material Table 3).

Analysis was performed on 78 patients who completed both the programme and 6-month review.

### Baseline characteristics

Baseline characteristics reported by patients on day 1 are illustrated in Table 1. From structured interview and assessments 50% reported predominately motor symptoms, 41% predominately non-epileptic episodes and 9% predominately sensory or cognitive symptoms. Furthermore, 81% had ‘any’ (at least one) motor symptom, 65% had ‘any’ sensory symptoms. From the PHQ15 somatic symptom scores, the highest reported somatic symptom categories were tired/low energy (94%), pain (69%), trouble sleeping (82%) and headaches (82%). The most prevalent education category was lower secondary school (GCSE grade C equivalent and below) (see Supplementary material for glossary).

**Table 1:** Patient-reported symptoms and characteristics at baseline given in structured interview

Demographics	*n* (%)
Age (SD)	42.6 years (13.5), range 19 to 76 years
Gender frequency	Female 60 (77), Male 18 (23)
Mean symptom duration (SD)	6.5 years
Age at symptom onset	36 years
Not working due to symptoms	66 (85)
On illness-related benefits	59 (66% of females and 50% of males)
Education (highest level attained)	
Primary	3 (3.8)
Secondary lower	28 (35.4)
GCSE, O level, CSE	18 (22.8)
Further education	3 (3.8)
HND,NVQ, BTEC	4 (5)
Secondary higher A levels	19 (24.1)
University degree	19 (24.1)
University masters	2 (2.5)
University doctorate	0 (0)
Predominant symptom	
Functional motor	39 (50)
Non-epileptic episodes	32 (41)
Other (PPPD, cognition, sensory)	7 (9)
Any motor symptoms (weakness, gait, jerks, tremor, dystonia)	63 (81)
Any sensory symptoms (visual, hearing, pins and needles, numbness dizziness)	50 (65)
Number of patients bothered by somatic symptoms (from PHQ15), either ‘a little’ or ‘a lot’	
Tired or low energy	72 (94)
Pain (arms, legs, joints)	69 (90)
Trouble sleeping	63 (82)
Headaches	63 (82)
Back pain	62 (81)
Constipation, loose bowel, diarrhea	54 (70)
Heart pounding/racing	52 (68)
Nausea, gas, indigestion	51 (66)
Dizziness	50 (65)
Stomach pain	48 (62)
Shortness of breath	48 (63)
Chest pain	35 (45)
Fainting spells	34 (44)
Menstrual cramps	30 (39)
Pain/problems during sex	22 (29)

Table 2 shows the frequencies over three time points of depression, anxiety, social anxiety and self-confidence by patient-reported questionnaires. At the start, although only 14% self-reported feeling low in mood in their initial assessment interview, when they were further assessed with the PHQ9, 92.3% met the IAPT depression caseness threshold of ≥ 10 [35], indicating at least a moderate depressive episode. Furthermore, severe depression was indicated by 21.8% of patients, moderately severe depression by 29.5%, moderate depression by 23.1% and mild levels were reported by 17.9%. Only 7.7% reported no depression as assessed by the PHQ9.

**Table 2:** Mental well-being—frequency analyses over three time points measured by patient-reported questionnaires

	Admission *n* (%)	Discharge *n* (%)	6months^e^ *n* (%)
PHQ9^a^			
None (0–4)	6 (7.7)	15 (19.2)	17 (21.8)
Mild (5–9)	14 (17.9)	22 (28.2)	18 (23.1)
Moderate (10–14)	18 (23.1)	28 (35.9)	27 (34.6)
Moderate-severe (15–19)	23 (29.5)	6 (7.7)	8 (10.3)
Severe (20–27)	17 (21.8)	7 (9)	8 (10.3)
GAD7^b^			
None (0–5)	18 (23.1)	40 (51.3)	35 (44.9)
Mild (6–10)	23 (29.5)	17 (21.8)	13 (16.9)
Moderate (11–15)	18 (23.1)	9 (11.5)	15 (19.5)
Severe (16–21)	19 (24.4)	12 (15.4)	14 (17.2)
SPIN^c^			
None (0–20)	32 (41)	40 (51.1)	46 (59)
Mild (21–30)	13 (16.7)	18 (23.1)	14 (17.9)
Moderate (31–40)	19 (24.4)	10 (12.8)	9 (11.5)
Severe (41–50)	6 (7.7)	5 (6.4)	5 (6.4)
Very severe (above 50)	7 (9)	5 (6.4)	4 (5.1)
Rosenberg ^d^			
Very low (0–10)	22 (28.2)	10 (12.8)	8 (10.3)
Low (11–15)	19 (24.4)	20 (25.6)	22 (28.2)
Moderate (16–20)	20 (25.6)	25 (32.1)	28 (35.9)
High (21–25)	13 (16.7)	18 (23.1)	11 (14.1)
Very high (26–30)	4 (5.1)	5 (6.4)	9 (11.5)

^a^PHQ9 Caseness ≥ 10

^b^GAD7 Caseness ≥ 8

^c^SPIN Caseness ≥ 19

^d^Rosenberg 0–14 indicates low self-esteem

^e^One missing data in this timepoint (*n* = 77)

Regarding anxiety, on admission, 23.1% reported no anxiety and 71% met the IAPT caseness threshold for anxiety of ≥ 8 when measured by the GAD 7 [35]. Of the 76.9% who reported anxiety, 24.4% was of a severe degree, 23.1% moderate and 29.5% mild.

The SPIN indicated that at baseline 59% met the IAPT caseness threshold ≥ 19 for social anxiety with features of fear, avoidance and physiological arousal. Low self-esteem at baseline was present in 50% of patients as measured by the Rosenberg scale.

Below, we explore whether the treatment was perceived as beneficial and then detail the evidence for its efficacy.

### VAS benefit of programme

The patient rated average benefit of programme visual analogue score was 90%.

### Somatic symptoms and mental well-being indices

The analyses (Table 3) yielded significant improvements in somatic symptoms (PHQ15), depressive (PHQ9) and anxiety (GAD7) symptoms. The effect sizes (Kendall’s W) were small for all measurements, particularly for GAD7 and PHQ15. Dunn-Bonferroni post hoc tests were carried out to understand the nature of these improvements. The analyses yielded a significant result between the median scores obtained at discharge compared to admission scores and 6-month follow-up and admission. Non-significant results were obtained between the 6-month follow-up and discharge. This pattern suggests that the improvements in these outcome measures were obtained at discharge and remained stable at follow-up.

**Table 3:** Median scores and non-parametric repeated measures analyses of variance over the three time points (admission, discharge and 6-month follow-up)

	*N*				Statistical significance			
		Admission ^1^	Discharge^1^	6 months^1^	Friedman’s ANOVA		Kendall’s W^3^ (%)	Dunn’s pairwise tests^4^
		(1)^2^	(2)^2^	(3)^2^	(χ^2^, *p* value)			
Somatic symptoms								
PHQ15^a^	77	15 (8)	13 (8)	12 (8)		18.1, *p* < 0.001	11.7	2 < 1^***^; 3 < 1^***^; 2 = 3
Mental well-being								
PHQ9^b^	78	15 (10)	10 (9)	10 (8)		33.2, *p* < 0.001	21.3	2 < 1^***^; 3 < 1^***^; 2 = 3
GAD7^c^	77	10 (9)	5 (9)	7 (12)		14.9, *p* < 0.001	9.7	2 < 1^**^; 3 < 1^**^; 2 = 3
SPIN^d^	77	25 (26)	20 (23)	16 (20)		4.3, *p* > 0*.*05	2.8	Not applicable
Rosenberg self-esteem^e^	78	14.5 (10)	17.5 (7)	17 (7)		12.6, *p* < 0.005	8	2 > 1^**^; 3 = 1; 3 = 2
Functionality								
WSAS^f^ (disability)	77	20.5 (17)	15 (13)	14 (13)		23.7, *p* < 0.001	15.4	2 < 1^***^; 3 < 1^***^; 2 = 3
COPM (performance)^g^	78	3.2 (1.8)	5.5 (2.8)	5.89 (3.4)		96.9, *p* < 0.001	62.1	2 > 1^***^; 3 > 1^***^; 2 = 3
COPM (satisfaction)^h^	78	2.55 (2.1)	5.6 (3.2)	6.1 (3.3)		89.1, *p* < 0.001	57.2	2 > 1^***^; 3 > 1^***^; 2 = 3
Health and social functioning								
HONOS^i^	78	15 (4)	11 (5)	9 (6)		105.186, *p* < 0.001	67.4	2 < 1^***^; 3 < 1^***^; 3 < 2^**^
Health status								
EQ-5D-5L VAS^j^	78	50 (25)	60 (25)	59 (25)		21.414, *p* < 0.001	13.7	2 > 1^***^; 3 > 1^*^; 2 = 3

^1^As data were not normally distributed, the values in the time point columns are the median and interquartile range (Median (IQ range)

^2^Time points were assigned numbers to summarise the results of the Dunn’s pairwise post hoc test

^3^Kendall’s W uses the Cohen’s interpretation guidelines of 0.1 (small effect), 0.3 (moderate effect) and above 0.5 (strong effect)

^4^All reported *p* values are after Bonferroni adjustments

^a^Score range = 0–30. Higher score represents worse somatic symptoms; minimal 0–4, low 5–9, medium 10–14, high 15–30

^b^Score range = 0–27. Higher score indicates worse depressive symptoms: none 0–4, mild 5–9, moderate 10–14, moderate severe 15–19, severe 20–27

^c^Score range = 0–21. Higher score indicates greater anxiety: none 0–5, mild 6–10, moderate 11–15, severe 16–21

^d^Score range = 0–68. Higher score indicates worse social phobia symptoms: ≤ 20 none, 21–30 mild, 31–40 moderate, 41–50 severe, ≥ 51

^e^Score range = 0–30. Higher score indicates higher self-esteem

^f^Score range = 0–40. Higher score indicates greater impairment

^g^Score range = 1–10. Higher score indicates better performance

^h^Score range = 1–10. Higher score indicates higher satisfaction

^i^Score range = 0–48. Higher score indicates greater impairment: very severe

^j^Score range = 0–100. Higher score indicates better health

*p < 0.05

**p < 0.005

***p < 0.001

An improvement in self-esteem was noted between discharge and admission. This effect disappeared at the 6-month follow-up and the effect sizes for these measurements were small. No significant results were obtained for the social anxiety scale (SPIN) despite a consistent reduction across time points and an overall nine-point reduction between admission and 6 months. This may be due to the heterogenous nature of the group.

### Health and social functioning

Analyses of the clinician-rated HoNOS yielded a significant improvement in overall impairment, with a big effect size (67.4%). Dunn-Bonferroni post hoc testing revealed significant results across all comparisons so that the reported median scores were better between the 6-month follow-up and discharge (Table 3).

On admission, median disability level as measured by WSAS was 20.5, indicating severe impairment in function and by IAPT estimations, suggestive of moderately severe psychopathology. Scores dropped on discharge to 15 (moderate impairment) and reduced further at 6-month follow-up to 14 (moderate impairment). The effect size of the model was small (15.4%) (Table 3).

### Functional performance

The Canadian Occupational Performance Measure (COPM) results indicated that both performance and satisfaction ratings on the patients’ self-selected priority occupation areas improved so that median scores were significantly higher at discharge compared to admission and remained stable at the 6-month follow-up. The effect size obtained was large for both measurements (Table 3).

### Health status

The results of the EQ-5D-5L assessing health status overtime are summarised in Table 4. For comparison, column 6 illustrates the EQ5D-5L value set for the general population in England as reported in 2015 [36]. This demonstrates that on admission, our patient group with FNSD has worse values for mobility, self-care, usual activities, pain/discomfort and anxiety/stress than the general population values.

**Table 4:** Frequencies reporting levels 1–5 by dimension of the EQ-5D-5L, over the three time points (admission, discharge and 6-month follow-up)

EQ-5D-5L	Problem	Admission *n* (%)	Discharge *n* (%)	6 months *n* (%)	EQ5D-5L value set for England 2015 (for comparison) *n* (%)
Mobility (*n* = 78)	Level 1	9 (11.5)	19 (24.4)	19 (24.4)	737 (74)
	Level 2	20 (26)	25 (32.1)	19 (24.4)	113 (11.4)
	Level 3	26 (33)	21 (26.9)	26 (33.3)	80 (8)
	Level 4	18 (23)	10 (12.8)	10 (12.8)	58 (5.8)
	Level 5	5 (6.4)	3 (3.8)	4 (5)	8 (0.8)
Self-care (*n* = 78)	Level 1	27 (34.6)	38 (48.7)	37 (47.4)	904 (90.8)
	Level 2	28 (35.9)	22 (28.2)	24 (30.8)	35 (3.5)
	Level 3	18 (23)	14 (17.9)	14 (17.9)	36 (3.6)
	Level 4	5 (6.4)	4 (5.1)	2 (2.6)	15 (1.5)
	Level 5	0 (0)	0 (0)	1 (1.28)	6 (0.6)
Usual activity (*n* =78)	Level 1	1 (1.3)	10 (12.8)	7 (9)	760 (76.3)
	Level 2	13 (16.7)	20 (25.6)	25 (32)	107 (10.7)
	Level 3	32 (41)	35 (44.9)	34 (44)	68 (6.8)
	Level 4	25 (32)	9 (11.5)	10 (12.8)	49 (4.9)
	Level 5	7 (9)	4 (5)	2 (2.6)	12 (1.2)
Pain/discomfort (*n* = 78)	Level 1	3 (3.8)	6 (7.7)	6 (7.7)	582 (58.4)
	Level 2	19 (24.4)	25 (32.1)	15 (19.2)	226 (22.7)
	Level 3	25 (32)	24 (30.8)	35 (45)	104 (10.4)
	Level 4	23 (29.5)	20 (25.6)	21 (27)	71 (7.1)
	Level 5	8 (10.3)	3 (3.8)	1 (1.28)	13 (1.3)
Anxiety/stress (*n* = 78)	Level 1	10 (12.8)	17 (21.8)	17 (22)	757 (76)
	Level 2	23 (29.5)	25 (32)	21 (27)	137 (13.8)
	Level 3	26 (33.3)	24 (30.8)	28 (36)	73 (7.3)
	Level 4	14 (17.9)	6 (7.7)	7 (9)	20 (2)
	Level 5	5 (6.4)	6 (7.7)	5 (6.4)	9 (0.9)

Level 1—no problem

Level 2—slight problems

Level 3—moderate problems

Level 4—severe problems

Level 5—unable to do/extreme problems

Further analysis indicates an increase in prevalence in all the domains for level 1 (no problem) between the admission and 6-month follow-up. This is accompanied by a decrease in prevalence for level 4 and 5 (severe problem, extreme problem, respectively) across all the domains.

**Fig. 1:**
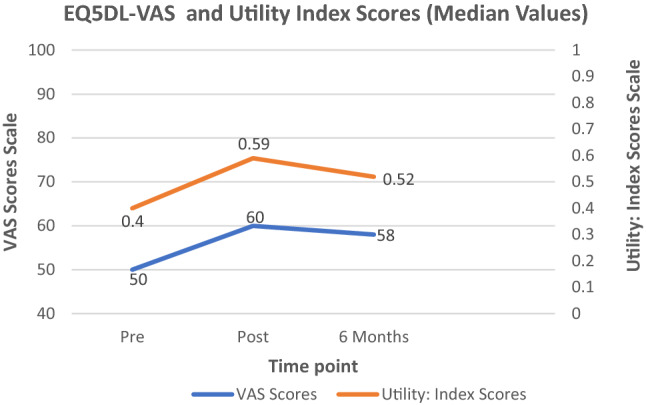
EQ-5D-5L Vas scores over time and EQ-5D-5L Utility Index scores over time (median values). Where EQ-5D-5 VAS score of 100 is ‘best imaginable health’ and 0 is ‘worse imaginable health’ and an EQ5DL Utility Index score of 1.0 represents full health and 0 is death

These findings are consistent with the overall health score results (EQ-VAS) (see Table 3 and Fig. 1), which indicate an increase in the overall health of patients between admission and discharge and the 6-month follow-up and admission time points. The effect size was small (13.7%). Figure 1 also illustrates an increase in utility from EQ-5D-5L Index values over the three time frames suggesting an improvement in health status (where 0 = death and 1 = full health).

**Fig. 2:**
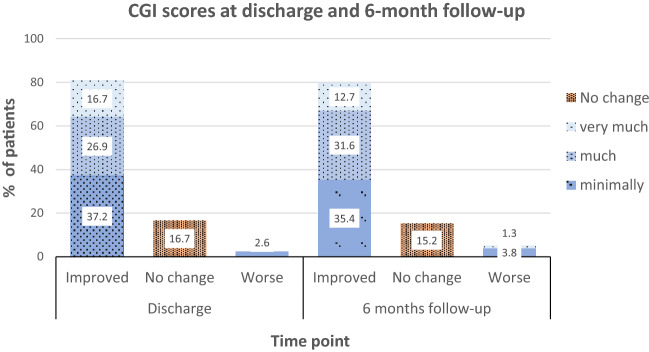
CGI (Clinical Global Improvement) collapsed scores at discharge and 6-month follow-up

Clinical improvements at discharge were broadly maintained at 6-month follow-up. Figure 2 shows this comparison by combining scores into two categories: improved categories (1–3); and no change (4) with worse categories (5–7). 80% rated themselves better at the end of 5 weeks which was sustained at 80% at the 6-month follow-up.

## Discussion

### Triage and generalisability

This study focuses on a selected group of FNSD patients who were: referred to a tertiary service (either due to complexity, locality or lack of availability of treatment locally), motivated to attend, ready and suitable to participate in a neuropsychiatry-led programme and had completed outcome measures on admission, discharge and 6-month review.

### Main findings

We have found that a 5-week MDT-based outpatient programme for a range of functional neurological symptoms with high baseline levels of somatic symptoms (pain, fatigue, etc.), anxiety and depression, is associated with statistically improved scores on a range of outcome measures and improvements from admission to discharge are largely sustained at 6-month follow-up. There was a high acceptance of this neuropsychiatry-led outpatient-based MDT treatment programme as indicated by the patient-rated VAS benefit of programme at 90%, which although not validated, has face validity.

### Co-morbidities

Notably, the rate and degree of depression and anxiety in this population, remain high and persistent despite improvements. This is consistent with previous findings of high rates in tertiary centres which have been associated with persistence of symptoms and associated with poorer long-term prognosis [8, 10]. Discrepancies between self-report and questionnaire data indicate that questionnaire evaluation is more systematic and may be a reliable way of assessing co-morbidities over different time points. Pain and fatigue are the highest somatic symptoms reported at baseline (94% and 90%, respectively). They should be expected and can be addressed through this MDT approach to FNSD through education and modification of inefficient or maladaptive behaviours/states, e.g., movements, boom-bust, depression, anxiety and medication reviews. However, where they are seen as separate to FNSD and are not seen as potentially modifiable through the outlined therapeutic approach and are of a degree that would significantly impair participation prior to being addressed (e.g., non-attendance, side effects from high-dose opioids), then exploring alternative approaches such as pain management clinics (e.g., reviewing appropriateness of medication and psychological factors) may be a helpful starting point. These factors should be considered and discussed early on to maximise an individual’s likelihood of benefitting from this time-limited programme.

Patients with FNSD or NES often struggle to identify and report co-morbidities and sometimes the presence of FNSD can act as a barrier to accessing both appropriate diagnosis and treatment within local services. If co-morbidities are not identified, addressed or prove difficult to treat within treatment programme time frames, they may impact on outcomes and perpetuate chronicity.

### Relevance

This outpatient programme is relevant as it provides a potentially cheaper method of delivering a multidisciplinary model of care to patients with a range of functional neurological symptoms in 10 days spread over 5 weeks. It can be used as an alternative to potentially more costly inpatient care which can be reserved for patients with nursing needs or specific interventions not otherwise deliverable in an outpatient setting.

As the programme aims to integrate education and symptom management techniques into daily function, this is reinforced by not staying in hospital and negotiating the environment beyond hospital on a daily basis. The 6-month follow-up allows a period of consolidation and patients can troubleshoot any difficulties that have arisen.

The neuropsychiatry-led outpatient programme has high rates of patient acceptability and can be less disruptive for individuals and families, those who are working and those who are uncomfortable in inpatient settings.

### Comparison with other programmes

Compared to general populational norms for health status and health-related quality of life [36], our patients with FNSD, despite showing sustained improvements following treatment, remain to some degree, impaired. This is consistent with the the literature [5].

Other outpatient-based approaches have focused on particular treatment modalities for specific symptoms, e.g., physiotherapy for functional motor disorders [37] or CBT for non-epileptic seizures [14] and reported lower co-morbid anxiety and depression levels on admission. Another recent approach is a group-based day programme rather than individualised sessions with different treatment modalities. At this point, there is no data for comparison.

The CGI results of our current 5-week outpatient programme are broadly comparable to previously published results from our 4-week inpatient programme which has additional nursing input for those requiring it ^(21)^. Comparison of CGI scores between the two programmes with collapsed scores is as follows: outpatient programme endorsement of CGI categories: ‘improved/better’ 80% on discharge and 80% at 6-month follow-up; ‘no change’ 17% on discharge and 15% at 6-month follow-up and ‘worse’ 3% at discharge and 5% at follow-up. By comparison, the inpatient endorsement of CGI categories was: ‘improved/better’ 72% on discharge and 67% at 1-year follow-up; ‘no change’ 22% on discharge and 17% at 1-year follow-up and ‘worse’ was endorsed by 5% on discharge and 17% at 1-year follow-up.

Furthermore, the mean depression scores of the inpatient programme were 15.8 on admission (moderately severe) reducing to 13.3 on discharge (moderate) as measured by the HADs. By comparison, the outpatient mean depression scores on admission were 14 (moderate) and on discharge 10 (mild) as measured by the PHQ9. Of note, these different measures of depression by HADS and PHQ9 differ in their rating of severity with a possibility that PHQ9 categorises a higher proportion of people as severe [38]. Nevertheless, the measures used for the outpatient MDT programme were intended to map onto those used by IAPT, a UK nationwide service, delivering psychological therapies locally. This was to facilitate onward referral locally for identified co-morbidities including depression and anxiety. Differences between the two studies are that a small proportion of the inpatient population at the time of the 2014 study, were likely to have had a higher level of severity requiring nursing input and the follow-up period was at a year and was by telephone rather than face to face as on the outpatient programme.

### Limitations

We acknowledge the limitations of the current study focused on a selected group. Physical outcome measures (e.g., 10MTW) although performed for subgroups, were not included in these analyses due to the widespread heterogeneity of symptoms. This is a pragmatic programme, within the National Health Service and treats a heterogenous range of FNSD both between and within individuals with a range of co-morbidities. There has been no use of a placebo or comparison group which would better assess the relationship between the intervention and improved outcomes. Further studies with different designs are required to assess which components of the programme have led to which gains or to analyse the effect of factors such as symptom duration on outcome.

### Conclusion

An outpatient neuropsychiatry-led MDT programme for FNSD can serve as a potential alternative to inpatient care for patients who have fewer or no nursing needs, for those whose preference is an outpatient setting and for those whose trajectory is chronic and for whom intermittent input with time at home to consolidate gains between sessions, is preferable to continuous input on a ward. Focus is on education and ultimately better self-management.

## Electronic supplementary material

Below is the link to the electronic supplementary material.

Supplementary file1 (DOCX 32 kb)
