# Supplementary material for: Outcomes of a 5-week individualised MDT outpatient (day-patient) treatment programme for functional neurological symptom disorder (FNSD)
Source: J Neurol. 2020 May 14;267(9):2655–66. doi: 10.1007/s00415-020-09874-5 (PMC7419475; doi:10.1007/s00415-020-09874-5)
Supplement: Supplementary file 1 — Supplementary file1 (DOCX 32 kb) [file 415_2020_9874_MOESM1_ESM.docx]

**Supplementary material**

**Table 1 - Outcomes of MDT assessment clinics**

| n = 277 | % = 100 | Outcome of MDT Assessment clinics 15 month period with criteria |
| --- | --- | --- |
|  |  |  |
| 47 | 17% | Did not attend and were discharged |
| 60 | 22% | Inpatient (need for nursing and/or speech and language therapy) |
| 109 | 39% | Outpatient (able to manage in a hotel) |
| 16 | 6% | CBT (pure NES, low physiotherapy / occupational therapy needs) |
|  | 16% | Other |
| 12 | 4% | Not appropriate time for patient (caring for others, work) |
| 8 | 3% | Non agreement with diagnosis |
| 5 | 2% | Pain management referral more appropriate |
| 6 | 2% | Improved - no further input sought |
| 3 | 1% | Referred to longer programme due to complexity |
| 3 | 1% | Acute mental health stabilisation required |
| 2 | 0.7 | Physiotherapy referral locally |
| 2 | 0.7 | Psycho-social support sought |
| 2 | 0.7 | Duplication of other MDT programmes patient referred to |
| 2 | 0.7 | Unable to identify goals |

**Table 2 - Outcome measures**

| Measure | Description |
| --- | --- |
| PHQ15 | Patient reported screening tool for somatisation syndromes. Score range = 0–30. Higher score represents worse somatic symptoms; minimal 0-4, low 5-9, medium 10-14, high 15-30. |
| PHQ9  Physical Health Questionnaire | Patient reported measure of depression. 0-4 none, 5-9 mild,10-14 moderate, 15-19 moderate-severe, 20-27 severe. A score of >9 is indicative of moderate-severe depression.  IAPT caseness threshold > 10. Reliable change > 6. |
| GAD7  Generalised Anxiety Disorder Scale | Patient reported measure of generalised anxiety disorder. 0-5 none, 6-10 mild, 11-15 moderate, 16-21 severe. IAPT caseness threshold >8. Reliable change >4 . |
| SPIN Social phobia inventory | Patient reported measure for social anxiety (social phobia) over the last week. < 20 none, 21-30 mild, 31-40 moderate, 41-50 severe, >51 very severe. > 19. Reliable change > 10. |
| Rosenberg's Self-Esteem | Patient reported measure of global self-worth by measuring both positive and negative feelings about the self. 0-14 low self-esteem, 15-25 normal self-esteem, 25-30 high self-esteem. |
| WSAS  Work and Social Adjustment Scale | A patient reported measure designed to scale patients perceived functional impairment resulting from a health problem. Covers domains of work, home management, social and private leisure activities and relationships with others. Scores range from 0 to 40 with lower scores indicating better adjustment. > 20 (out of 40) = impaired (with work) > 16 (out of 32) impaired (without work). 0-9 low impairment10-19 = mod impairment. 20-40 severe impairment. |
| COPM  Canadian Occupational Performance Measure | An individualised, client-centred outcome measure designed to capture a client's self-perception of performance and satisfaction with their performance in everyday living, over time. Patients set their own priority occupational areas aided by an occupational therapist. A change of 2 points between to time points is considered clinically significant. |
| HONOS  Health of the Nation Outcomes Scale | Clinician rated 12 point scale – covering a wide range of health and social domains- including  psychiatric symptoms, physical health, functioning, relationships and housing.  Intended for repeated measures to assess change. |
| EQ-5D-5L | A patient reported measure of health status and health related quality of life. Can generate societal values (utilities) which can be used in economic evaluations. It is a descriptive system comprising 5 dimensions: mobility, self-care, usual activities, pain/discomfort and anxiety/depression. Each dimension has 5 levels: no problem, slight problems, moderate problems, severe problem, unable to do / extreme problems. The respondent is asked to indicate his/her health state by ticking the most appropriate statement in each of the 5 dimensions. |
| EQ VAS  Visual Analogue Scale | Patient-rated health on a vertical, visual analogue scale where the endpoints are labelled ‘best imaginable health state’ and ‘worst imaginable health state’. This information can be used as a quantitative measure of health outcome as judged by the individual respondents. |
| Visual analogue scale of Benefit of programme | Patient rated outcome measuring benefit of programme. |
| CGI  Clinical Global Improvement scale | Patient reported perception of symptom improvement. 1. Very much improved, 2. Much improved, 3. Minimally improved, 4. No change, 5. Minimally worse, 6. Much worse  7. Very much worse. |

**Table 3 - Comparison of the 19 patients excluded from study as they did not attend the 6th month review with the 78 included in the study.**

|  |  | **DNA 6M review**  **so excluded from study** | | | **Attended 6M review so included in study** | | |
| --- | --- | --- | --- | --- | --- | --- | --- |
|  |  | *n* | % |  | *n* | % |  |
| Total |  | 19 |  |  | 78 |  |  |
|  | F | 12 | 63% |  | 60 | 77% |  |
|  | M | 7 | 37% |  | 18 | 23% |  |
| Predominant Symptom | FMD | 12 | 63% |  | 39 | 50% |  |
|  | NES | 4 | 21% |  | 32 | 41% |  |
|  | Sensory or Cognitive | 3 | 16% |  | 7 | 9% |  |
|  |  | **N** (19) | **Mean** | **Severity rating** | N (78) | **Mean** | **Severity**  **rating** |
| PHQ 15 admission |  |  | 14 | medium |  | 15 | high |
| PHQ 15 discharge |  |  | 11 | medium |  | 13 | medium |
| PHQ9 admission |  |  | 14 | moderate |  | 15 | moderate-severe |
| PHQ9 discharge |  |  | 10 | moderate |  | 10 | moderate |
| GAD7 admission |  |  | 8 | mild |  | 10 | mild |
| GAD 7 discharge |  |  | 8 | mild |  | 5 | none |
| HONOS admission |  |  | 14 |  |  | 15 |  |
| HONOS discharge |  |  | 12 |  |  | 11 |  |
| SPIN admission |  |  | 20 | none |  | 25 | mild |
| SPIN discharge |  |  | 20.5 | none |  | 20 | none |
| Rosenberg Self-esteem admission |  |  | 17 | normal |  | 14.5 | low |
| Rosenberg Self-esteem discharge |  |  | 19 | normal |  | 17.5 | normal |
| WSAS admission |  |  | 18 | moderate |  | 20.5 | Severe |
| WSAS discharge |  |  | 14 | moderate |  | 15 | moderate |
| COPM Perf Admission |  |  | 3.75 |  |  | 2.55 |  |
| COPM Perf Discharge |  |  | 6.75 |  |  | 5.5 |  |
| COPM Satisf Admission |  |  | 3.50 |  |  | 2.55 |  |
| COPM Satisfaction Discharge |  |  | 7.25 |  |  | 5.6 |  |
| EQ5DL VAS Health status - admission |  |  | 53 |  |  | 50 |  |
| EQ5DL VAS Health status - discharge |  |  | 67 |  |  | 60 |  |
| VAS benefit of programme |  |  | 81 |  |  | 90 |  |

**Glossary**

- GSCE – General certificate of Secondary Education - record of achievement at the age of 16, in place of a leaving certificate at schools in England, Wales and Northern Ireland.
- BTEC - The Business and Technology Education Council is a provider of [secondary](https://en.wikipedia.org/wiki/Secondary_education_in_the_United_Kingdom) [school leaving qualifications](https://en.wikipedia.org/wiki/School_leaving_qualification) and [further education](https://en.wikipedia.org/wiki/Further_education) qualifications in [England](https://en.wikipedia.org/wiki/England), [Wales](https://en.wikipedia.org/wiki/Wales) and [Northern Ireland](https://en.wikipedia.org/wiki/Northern_Ireland).
- HND - Higher National Diploma) is a two year course, equivalent to the first two years of university
- NVQs - National Vocational Qualifications were work-based awards in England, Wales and Northern Ireland achieved through assessment and training. The regulatory framework supporting NVQs was withdrawn in 2015 and replaced by the Regulated Qualifications Framework (RQF), although the term "NVQ" may be used in RQF qualifications if they are based on recognised occupational standards, work-based and/or simulated work-based assessment and where they confer occupational competence.
- PPPD – Persistent Postural Perceptual Dizziness
